# Preparation of Polyphenylene Ring Derivative Dyes with Wide Wave Absorption Properties and Their Performance Study

**DOI:** 10.3390/molecules27175551

**Published:** 2022-08-29

**Authors:** Yuzhen Zhao, Xinhua Liu, Qing Li, Zhun Guo, Zemin He, Huimin Zhang, Cheng Ma, Jianjing Gao, Yang Zhao, Dong Wang

**Affiliations:** 1 Xi’an Key Laboratory of Advanced Photo-Electronics Materials and Energy Conversion Device, School of Electronic Information, Xijing University, Xi’an 710123, China; 2 Department of Materials Physics and Chemistry, School of Materials Science and Engineering, University of Science and Technology Beijing, Beijing 100083, China

**Keywords:** Azobenzene, click chemistry, nonlinear optics

## Abstract

Some conjugated benzene ring molecules were prepared using the Sonogashira reaction, and the molecules were post-functionally modified using click chemistry. The optical and electrical band gaps were measured using UV-VIS absorption spectroscopy and the three-electrode method, and the results of both were verified against each other to prove the accuracy of the characterization. In addition, the optical performances of the material were studied by z-scan; almost all materials exhibited good nonlinear optical properties and interconversion between saturable and anti-saturable absorption due to the invocation of click reagents.

## 1. Introduction

In recent years, many small molecules with excellent nonlinear optical properties have entered the limelight because of promising potential in a wide diversity of optical fields, for example, quantum communication, optical limiting, and information storage [1,2,3]. Organic small molecules have excellent properties such as diverse structural composition and wide space for property tuning, and the molecules can be designed to tune the actually desired properties. However, the preparation of current small-molecule conjugated materials is very complicated, and the chemical synthesis and purification are very tedious, which limits its development in some cases [4,5,6,7].

The emergence of click chemistry has effectively solved this problem. The click chemistry occurs in fast, side-reaction-free, near-100% yields and is easily purified [8,9]. The introduction of click chemistry into the preparation of nonlinear materials has led to the synthesis of a wide variety of organic small molecule nonlinear materials that express strong nonlinearity [10,11,12,13].

Traditionally, biphenyl-like molecules are widely used in pharmaceuticals, pesticides, dyes, liquid crystal materials [14,15,16,17], and also in the manufacture of fuels, engineering plastics, and high-energy fuels [18,19,20]. However, Biphenyl materials are rarely used as non-linear optical materials.

In this paper, a biphenyl-based conjugated structure molecule was constructed by Sonogashira coupling reaction and functionalized by click reagents, and the effects of different amounts and categories of click reagents on the molecular properties were investigated. The results indicate that these functionally modified molecules show a wide absorption. The type of click reagent has a strong influence on the molecular properties, compared to the symmetry of the molecule and the number of click reagents, which have less influence.

## 2. Preparation and Research

### 2.1. Preparation

**Figure 1:**
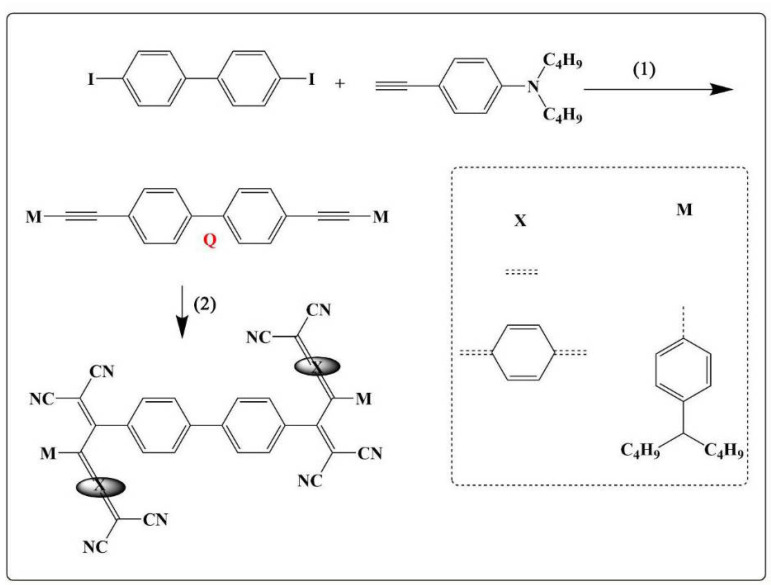

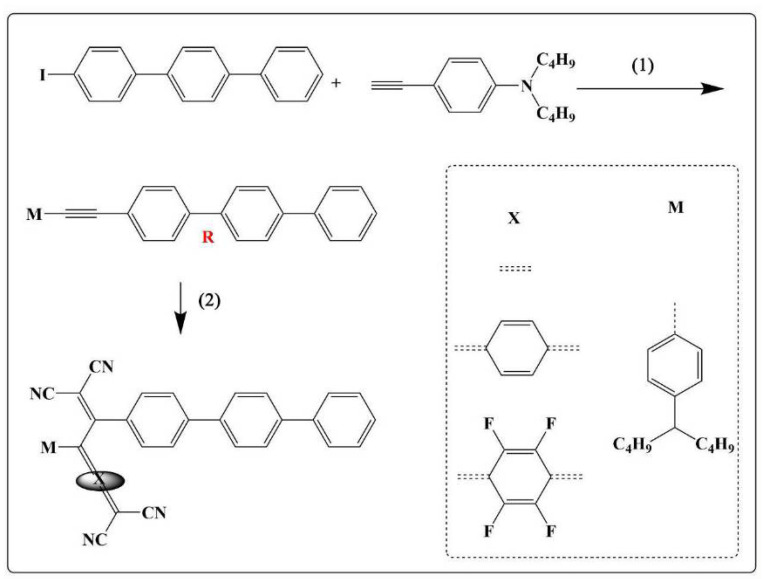
Total Synthesis route (**1**) PdCl_2_ (PPh_3_), CuI, DIPA, 60 °C, Ar, 36 h; (**2**) TCNE/TCNQ/F4-TCNQ, CH_2_Cl_2_, rt, 1 h.

The synthetic route of dibiphenyl derivatives is placed in Figure 1. Monobromobiphenyl and dibromobiphenyl and N, N-dibutyl−4-ethynylaniline are firstly used as raw materials and the target dibiphenyl derivatives are obtained in moderate yields by linking the two by Sonogashira coupling reaction. The dibiphenyl derivatives have two clickable triple bonds, to which different electron acceptors are introduced by a two-step click reaction, resulting in five molecules with different click structures. The yields of the different structural click products are presented in detail in the experimental section. Due to the difference in the number and structure of the introduced acceptor molecules, the polarity of all the obtained click products varies widely, which makes the separation of the click products very simple.

### 2.2. UV-Vis-NIR Spectro

**Figure 2:**
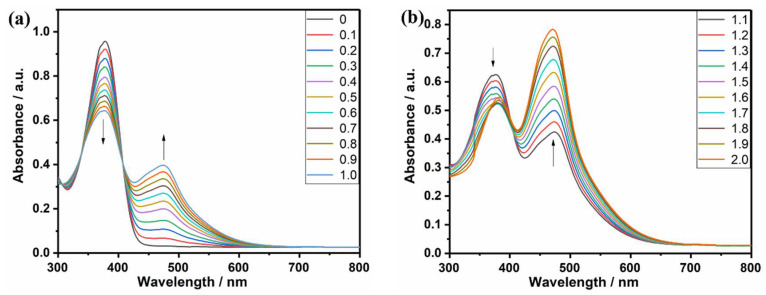
UV-VIS-NIR tracking spectrum of click response (**a**) TCNE (0–1.0 equivalent); (**b**) TCNE (1.0–2.0 equivalent).

To study the occurrence of click chemistry and its occurrence or not of side reactions, a click-track reaction is carried out in this paper using compound Q. The TCNE is titrated to Q in a fixed amount twenty times, and the progress of the reaction is monitored by UV-Vis spectroscopy. The titration experiments of 0–1 equivalents of TCNE are shown in Figure 2a. When 0.1 equivalents of TCNE are added to the CH_2_Cl_2_ solution, the absorption peak of the precursor (Q) at 376 nm started to decrease, and an absorption peak occurred in 470 nm, which represents the production of new compounds (Q-1, Q-11). As TCNE is continuously added to the solution, the absorption peak at 470 nm gradually increased until the absorption peak at 376 nm decreased to a minimum after the addition of 2 equivalents of TCNE, which indicates the complete conversion of Q to Q-11. In previous works [21,22,23,24,25,26], click chemistry is shown to be efficient and free of side reactions. In Figure 2a, the production of extinction dots at 348 nm and 405 nm proves that the click reaction is side-reaction free.

**Figure 3:**
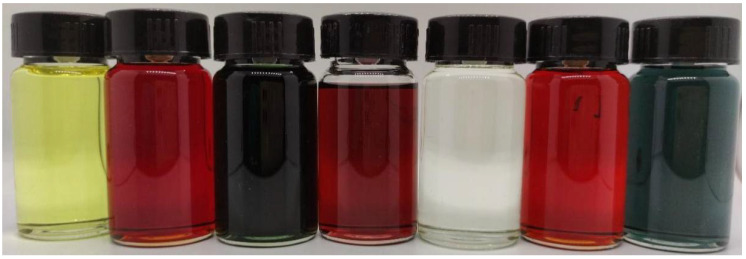
CH_2_Cl_2_ solution of click modification products of R and Q.

**Figure 4:**
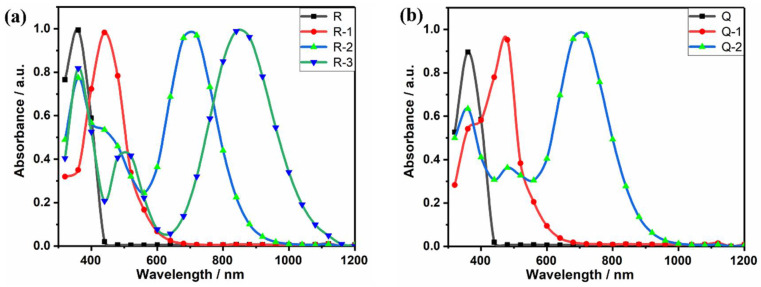
Absorption spectra of **(a**) R, R-1, R-2, R-3; (**b**) Q, Q-11, Q-22 in CH_2_Cl_2_ solution.

Figure 3 shows the CH_2_Cl_2_ solution of the click-modified products of R and Q. Figure 4a shows the UV-VIS-NIR spectroscopy of the compounds in the CH_2_Cl_2_ solution. The reaction of the light-yellow molecule R with one equivalent of the click reagents TCNE, TCNQ, and F4-TCNQ give brown R-1, green R-2, and purple R-3, respectively. In Figure 4b, the same redshift occurs in the click product of Q.

The main reason for the occurrence of the red-shift phenomenon is the introduction of click reagents that grow the conjugate length of the backbone and enhance the electron affinity [27,28]. The absorption peak of R-3 shows a larger redshift than R-1 and R-2, which is due to the introduction of strong electron-absorbing groups-CN and -F, which have a more pronounced effect of extending the conjugation length of the molecule. Compared with R, Q has two clickable triple bonds, so two acceptor molecules can be introduced. However, in Figure 4b, Q-11, Q-22 with two acceptor molecules introduced to show the same redshift of the maximum absorption peaks relative to R-1, R-2 with one acceptor molecule introduced, and the absorption peaks do not shift due to the different number of clickable reagents introduced, which indicates that the symmetry of the molecule has almost no effect on the position of the maximum absorption peak, and the type of click reagent has the greatest effect on redshift.

In conclusion, the introduction of click reagents can prolong the conjugation length of molecules, thus causing movement of the maximum peak, the degree of movement varies depending on the type of click reagents, resulting in a series of broadband absorbing dyes that can absorb different wavelengths.

### 2.3. Electrochemistry

**Figure 5:**
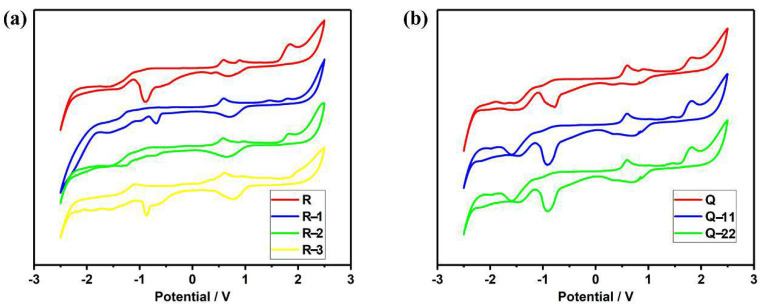
Cycle voltammogram of the molecular (**a**) R, R−1, R−2, R−3; (**b**) Q, Q−11, Q−22 in CH_2_Cl_2_/Bu_4_NPF_6_ at 298 K.

**Figure 6:**
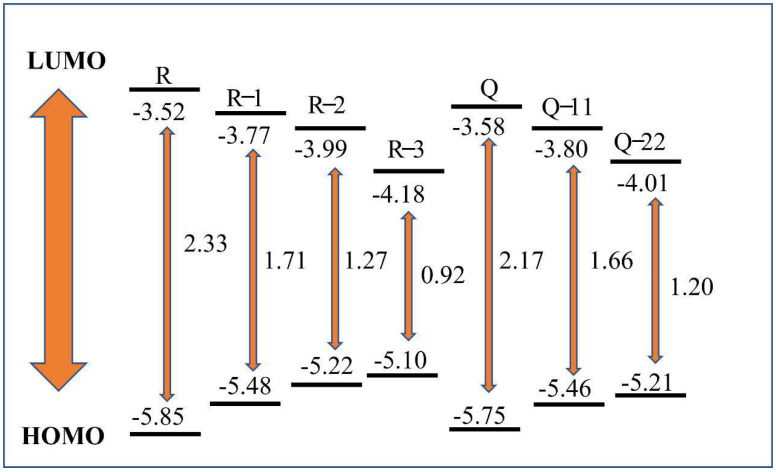
Electrical energy bands of benzene ring derivatives.

Electron off-domain is related to the nonlinear nature [25,29,30]. Figure 5 shows the cycle voltammogram and Figure 6 shows the energy level results of the compound. Table 1 also shows the initial redox potential. Electrical and optical energy bandwidths can be well matched, and both tend to decrease, thanks to the introduction of the acceptor molecules [31,32].

After the introduction of click reagents, the LUMO levels and band gaps of R-1, R-2, and R-3 showed a decreasing trend and the HOMO levels increased, and the band gap of R-3 decreased most significantly compared with R-1 and R-2, because of the groups -F and -CN in F4-TCNQ. Similarly, Q, Q-11, Q-22 show the same effect. By comparing R-1 and Q-11, R-2 and Q-22, it can be seen that the introduction of more click reagents in the molecule is less effective in reducing the bandgap, which can also indicate that the degree of conjugation of the molecule has a greater effect on the electrochemistry than the symmetry of the molecule. This is because the symmetry of the molecule has little effect on the degree of conjugation and does not change the conjugation structure, while the different kinds of click reagents introduced lengthen the conjugation length of the molecule, reduce the electron cloud density and the energy of the system, accelerate the intramolecular charge transfer, narrow the molecular band gap and make it easier for the leap.

### 2.4. Nonlinear Optics

The third-order nonlinear polarizability is one of the important parameters of the material [33]. The materials in this paper are tested under picosecond laser, some of them have poor nonlinear refractive properties, therefore, only the nonlinear absorption properties of the materials are investigated in this paper.

**Figure 7:**
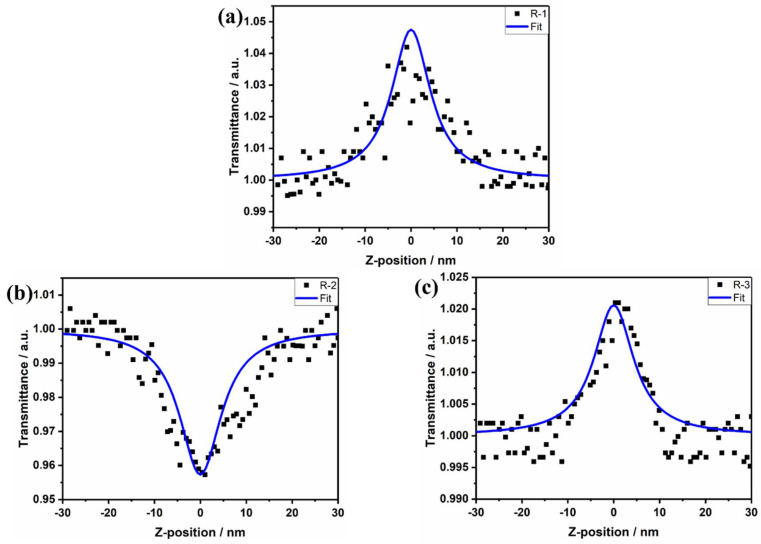
(**a**) R−1, (**b**) R−2, (**c**) R−3 Z-scan curves. The red line is the fitted data, and the scattered points are the Z-scan experimental data.

The Z-scan results of molecules are placed in Figure 7. No nonlinear optical properties are observed for molecule R which are shown in Table 2. Therefore, only R-1, R-2, R-3 are compared. It can be seen that after the click reaction, R-1 and R-3 have a peak in transmittance at the focal point and exhibit saturable absorption properties (SA), while R-2 has a valley in transmittance at the focal point and exhibits anti-saturation absorption properties (RSA), due to the alteration of the length of the molecular conjugate group as a result of the introduction of the click reagent, leading to a saturable absorption-trans-saturable absorption transition. The factors affecting the saturable to anti-saturable absorption transition are the absorption cross-section and the horizontal lifetime [34].

## 3. Materials and Methods

### 3.1. Material and Instrumental Characterization Equipment

All reagents were purchased from company sources and were ready for use. ^1^H NMR spectra of the materials were measured using a BRUKER AVANCEIIIHD400 with deuterated chloroform as the solvent and tetramethylsilane (TMS) as the internal standard. The UV absorption was measured by a JASCO V-570 spectrophotometer. The instruments for mass spectrometry analysis of materials were AB SCIEX MALDI-TOF/TOF 5800. Cyclic voltammetric curves of materials were measured using the CHI 660C instrument. Measurement of the response of nonlinear optical (NLO) characteristics uses a Z-scan technique (NLO-Z), where the laser pulse is chosen to be 21 ps.

### 3.2. Preparation of Materials

#### 3.2.1. Preparation of the Molecular R

N,N-dibutyl-4-ethynylaniline obtained from previous work [35] (1.6488 g, 0.0072 mol), 4-iodobiphenyl (0.6722 mg, 0.0024 mol), [PdCh (PPh_3_)] (0.00017 mol, 0.1206 g), CuI (0.00017 mmol, 0.034 g) were placed in the flask and 30 mL iPrNH was added, then shaken for 36 h at 60 °C. After the temperature dropped to room temperature, the solvent was cleared, and the separation technology column chromatography, the eluent is CH_2_Cl_2_, and the first crude purification was performed to remove the catalyst in the system. Then the product was purified by column chromatography and after distillation under reduced pressure, 566 mg of light-yellow solid product R was obtained, with a yield of 62%.

^1^H NMR (400 MHz, CDCl_3_, δ): 7.66–7.60 (m, 4H), 7.58 (s, 8H), 7.50–7.44 (m, 4H), 7.43–7.35 (m, 6H), 6.64–6.59 (m, 4H), 3.37–3.26 (m, 8H), 1.66–1.58 (m, 8H), 1.41 (m, 8H), 0.99 (m, 12H). IR (neat): 2964, 2937, 2873, 2202, 1590, 1526, 1376, 1200, 814, 773, 692, 542. MALDI-TOF-MS (dithranol): m/z: [MH]^+^ calcd. for C_28_H_31_N: 381.25, found: 382.76. Elemental analysis calcd. (%) for C_28_H_31_N (381.25): C 88.14, N 3.67, H 8.19, found: C 88.12, N 3.66, H 8.22.

#### 3.2.2. Preparation of the Molecular R-1

Compound R (152.4 mg, 0.4 mmol) was dissolved in 30 mL CH_2_Cl_2_ and TCNE (0.0512 g, 0.0004 mol) was added to it, then stirred for 40 min. After the reaction is over, the solvent was removed by distillation under reduced pressure, then a chromatographic column (the eluent is CH_2_Cl_2_) was used for purification to give R-1 (199.6 mg, 98%).

^1^H NMR (400 MHz, CDCl_3_, δ): 7.90–7.81 (m, 8H), 7.8–7.75 (m, 4H), 7.67–7.61 (m, 4H), 7.55–7.43 (m, 6H), 6.75–6.69 (m, 4H), 3.49–3.38 (m, 8H), 1.73–1.61 (m, 8H), 1.41 (m, 8H), 1.01 (m, 12H). IR (neat): 2959, 2869, 2220, 1603, 1490, 1340, 1209, 1181, 810, 769, 683, 578. MALDI-TOF-MS (dithranol): *m*/*z*: [M] calcd. For C_34_H_31_N_5_: 509.26, found: 509.69. Elemental analysis calcd. (%) for C_34_H_31_N_5_ (509.26): C 80.13, H 6.13, N 13.74, found: C 80.10, H 6.16, N 13.74.

#### 3.2.3. Preparation of the Molecular R-2

Compound R (152.4 mg, 0.4 mmol) was dissolved in 30 mL CH_2_Cl_2_ and TCNQ (0.0817 g, 0.0004 mol) was added to it, then stirred for 40 min. After the reaction is over, the solvent was removed by distillation under reduced pressure. Then a chromatographic column was used for purification to give R-2 (226.98 mg, 97%).

^1^H NMR (400 MHz, CDCl_3_, δ): 7.83–7.78 (m, 4H), 7.75–7.70 (m, 4H), 7.65–7.55 (m, 6H), 7.53–7.42 (m, 6H), 7.31 (m, 6H), 7.09 (m, 4H), 6.75–6.69 (m, 4H), 3.46–3.37 (m, 8H), 1.69–1.61 (m, 8H), 1.46–1.37 (m, 8H), 1.01 (m, 12H). IR (neat): 2969, 2933, 2869, 2197, 1586, 1399, 1345, 1177, 932, 833, 764, 733, 687, 542. MALDI-TOF-MS (dithranol): *m*/*z*: [M] calcd. for C_40_H_35_N_5_: 585.29, found: 585.96. Elemental analysis calcd. (%) for C_40_H_35_N_5_ (585.29): C 82.02, N 11.96, H 6.02, found: C 82.00, N 11.95, H 6.05.

#### 3.2.4. Preparation of the Molecular R-3

Compound R (152.4 mg, 0.4 mmol) was dissolved in 30 mL CH_2_Cl_2_ and F4-TCNQ (0.112 g, 0.0004 mol) was added to it, then stirred for 40 min. After the reaction is over, the CH_2_Cl_2_ was cleared by distillation, and then a chromatographic column was used for purification to give R-3 (259.1 mg, 98%), as a black solid.

^1^H NMR (400 MHz, CDCl_3_, δ): 7.77–7.72 (m, 4H), 7.72–7.67 (m, 4H), 7.66–7.61 (m, 4H), 7.54–7.44 (m, 6H), 7.40 (m, 4H), 6.89–6.84 (m, 4H), 3.59 (m, 8H), 1.76 (m, 8H), 1.50 (m, 8H), 1.05 (m, 12H). IR (neat): 2959, 2924, 2873, 2194, 1608, 1390, 1191, 1028, 964, 810, 778, 638, 533. MALDI-TOF-MS (dithranol): *m*/*z*: [M] calcd. for C_57_H_55_F_3_N_8_: 657.25, found: 657.77. Elemental analysis calcd. (%) for C_40_H_31_F_4_N_5_ (657.25): C 73.05, N 10.65, H 4.75, F 11.55, found: C 73.03, N 10.64, H 4.78, F 11.55.

#### 3.2.5. Preparation of the Molecular Q

N,N-dibutyl-4-ethynylaniline obtained from previous work [34] (1648.8 mg, 7.2 mmol), 4,4′-diiodobiphenyl (974.4 mg, 2.4 mmol), [PdCh (PPh_3_)] (120.6 mg, 0.172 mmol), CuI (34.0 mg, 0.178 mmol) were placed in the flask and 30 mL iPrNH was added, shaken for 36 h at 60 °C. After the temperature dropped to room temperature, the solvent was cleared, and the separation technology column chromatography, the eluent was CH_2_Cl_2_, and the first crude purification was performed to remove the catalyst in the system. Then the product was refined and after distillation under reduced pressure, 846.3 mg of light-yellow solid product Q was obtained, with a yield of 58%.

^1^H NMR (400 MHz, CDCl_3_, δ): 7.59 (d, J = 1.6 Hz, 4H), 7.46–7.33 (m, 2H), 6.66–6.55 (m, 2H), 3.37–3.27 (m, 4H), 1.59 (m, 4H), 1.39 (m, 4H), 0.99 (m, 6H). IR (neat): 2955, 2924, 2856, 2212, 1599, 1526, 1363, 1295, 1191, 1100, 1000, 928, 805, 533. MALDI-TOF-MS (dithranol): m/z: [MH]^+^ calcd. for C_44_H_52_N_2_: 608.41, found: 609.87. Elemental analysis calcd. (%) for C_44_H_52_N_2_ (608.41): C 86.79, N 4.60, H 8.61, found: C 86.77, N 4.60, H 8.63.

#### 3.2.6. Preparation of the Molecular Q-11

Molecular Q (0.243 g, 0.0004 mol) was dissolved in CH_2_Cl_2_ and TCNE (0.102 g, 0.0008 mol) was added, then stirred for 40 min. After the reaction was over, the solvent was removed by distillation under reduced pressure, and then a chromatographic column was used for purification to give Q-11 (331.7 mg, 96%). 

^1^H NMR (400 MHz, CDCl_3_, δ): 7.91–7.76 (m, 6H), 6.76–6.70 (m, 2H), 3.49–3.39 (m, 4H), 1.72–1.60 (m, 4H), 1.42 (m, 4H), 1.01 (m, 6H). IR (neat): 2969, 2938, 2873, 2206, 1603, 1485, 1422, 1345, 1191, 1000, 919, 814, 741, 545. MALDI-TOF-MS (dithranol): *m*/*z*: [M] calcd. for C_56_H_52_N_10_: 864.44, found: 864.84. Elemental analysis calcd. (%) for C_56_H_52_N_10_ (864.44): C 77.75, N 16.19, H 6.06, found: C 77.73, N 16.18, H 6.09.

#### 3.2.7. Preparation of the Molecular Q-22

Compound Q (0.243 g, 0.0004 mol) was dissolved in CH_2_Cl_2_ and TCNQ (0.166 g, 0.0008 mol) was added to it, then stirred for 40 min. After the reaction was over, the CH_2_Cl_2_ was cleared by distillation, then a chromatographic column for purification was used to give Q-22 (389.1 mg, 95%). 

^1^H NMR (400 MHz, CDCl_3_, δ): 8.14–7.75 (m, 4H), 7.75–7.49 (m, 4H), 7.05 (m, 2H), 6.71 (d, J = 9.2 Hz, 2H), 3.40 (m, 4H), 1.42 (m, 4H), 1.21 (m, 4H), 1.01 (m, 6H). IR (neat): 2964, 2918, 2873, 2202, 1571, 1404, 1340, 1187, 914, 846, 678, 532. MALDI-TOF-MS (dithranol): *m*/*z*: [MH]^+^ calcd. for C_68_H_60_N_10_: 1016.50, found: 1017.88. Elemental analysis calcd. (%) for C_68_H_60_N_10_ (1016.50): C 80.29, N 13.77, H 5.95, found: C 80.27, N 13.77, H 5.97.

## 4. Conclusions

Some benzene ring derivatives were prepared and systematically studied. UV-VIS-NIR spectroscopy and electrical testing showed that the introduction of click reagents increased the conjugate groups of the molecules, caused a movement of the maximum peak, while the energy bandwidth of the click products was reduced to different degrees. Z-scan tests showed that after clicking, the molecules all showed different nonlinear properties, and with the use of different click reagents, the molecules could undergo a transition between anti-saturation absorption and saturation absorption, yielding nonlinear materials with different properties.

## Figures and Tables

**Table 1:** Electrical properties of molecules.

Molecules	$\lambda_{end}{}^{a}$ (nm)	$E_{g}{\;}^{b}(\mathrm{eV})$	$E_{on}^{ox}{\;}^{c}(V)$	$E_{on}^{red\;}{}^{c}(V)$	HOMO (eV)	LUMO (eV)	$E_{g}{}^{\;d}(\mathrm{eV})$
R	412	3.00	1.25	−1.08	−5.85	−3.52	2.33
R-1	692	1.79	0.88	−0.83	−5.48	−3.77	1.71
R-2	982	1.26	0.62	−0.61	−5.22	−3.99	1.23
R-3	1188	1.04	0.50	−0.42	−5.10	−4.18	0.92
Q	442	2.80	1.15	−1.02	−5.75	−3.58	2.17
Q-11	686	1.80	0.86	−0.80	−5.46	−3.80	1.66
Q-22	1006	1.23	0.61	−0.59	−5.21	−4.01	1.20

^*a*^ UV-VIS spectrum cut-off absorption wavelength. ^*b*^ Optical energy bandwidth. ^*c*^ Initial oxidation potential and initial reduction potential (obtained by cyclic voltammetry characteristic curve). ^*d*^ Electrochemical band gaps.

**Table 2:** Nonlinear data for molecules.

Molecules	β × 10^−17^ (m/W)	χ^(3)^ × 10^−17^ (esu)
R-1	−30	6.45
R-2	30	6.45
R-3	−20	4.30
Q	15	3.23
Q-11	−33	7.09
Q-22	13	2.79

## Data Availability

The data presented in this study are available on request from the corresponding author.
